# Crystal Structure of Mesaconyl-CoA Hydratase from *Methylorubrum extorquens* CM4

**DOI:** 10.4014/jmb.2212.12003

**Published:** 2023-01-26

**Authors:** Jae-Woo Ahn, Jiyeon Hong, Kyung-Jin Kim

**Affiliations:** 1 Postech Biotech Center, Pohang University of Science and Technology, Pohang 37673, Republic of Korea; 2 Center for Biomolecular Capture Technology, Bio Open Innovation Center, Pohang University of Science and Technology, Pohang 37673, Republic of Korea; 3 School of Life Sciences, BK21 FOUR KNU Creative BioResearch Group, KNU Institute for Microorganisms, Kyungpook National University, Daegu 41566, Republic of Korea

**Keywords:** Mesaconyl-CoA hydratase, *Methylorubrum extorquens*, ethylmalonyl pathway, crystal structure

## Abstract

*Methylorubrum extorquens*, a facultative methylotroph, assimilates C_1_ compounds and accumulates poly-β-hydroxylbutyrate (PHB) as carbon and energy sources. The ethylmalonyl pathway is central to the carbon metabolism of *M. extorquens*, and is linked with a serine cycle and a PHB biosynthesis pathway. Understanding the ethylmalonyl pathway is vital in utilizing methylotrophs to produce value-added chemicals. In this study, we determined the crystal structure of the mesaconyl-CoA hydratase from *M. extorquens* (*Me*MeaC) that catalyzes the reversible conversion of mesaconyl-CoA to β-methylmalyl-CoA. The crystal structure of *Me*MeaC revealed that the enzyme belongs to the MaoC-like dehydratase domain superfamily and functions as a trimer. In our current *Me*MeaC structure, malic acid occupied the substrate binding site, which reveals how *Me*MeaC recognizes the β-methylmalyl-moiety of its substrate. The active site of the enzyme was further speculated by comparing its structure with those of other MaoC-like hydratases.

## Introduction

Various types of plastic materials, which are extensively used in modern civilization, are produced from petroleum. These petroleum-derived plastics result in serious pollution owing to their strong durability and resistance to degradation [1]. As an alternative for petroleum-derived plastics, bio-plastics derived from various microorganisms and plants have been in the spotlight. Among them, poly-hydroxyalkanoates (PHAs) have been researched for several decades due to their bio-compatibility, bio-degradability, and similar characteristics to traditional plastics [2-4]. Of more than 160 types of PHAs with different physical properties, poly-hydroxybutyrate (PHB) is the most common form of PHA, and is utilized in packaging, disposables, and medical supplies [1]. However, because PHB production through microbial fermentation is currently complex and expensive, it has become necessary to research other means of PHA biosynthesis using inexpensive carbon sources [5, 6].

**Fig. 1:**
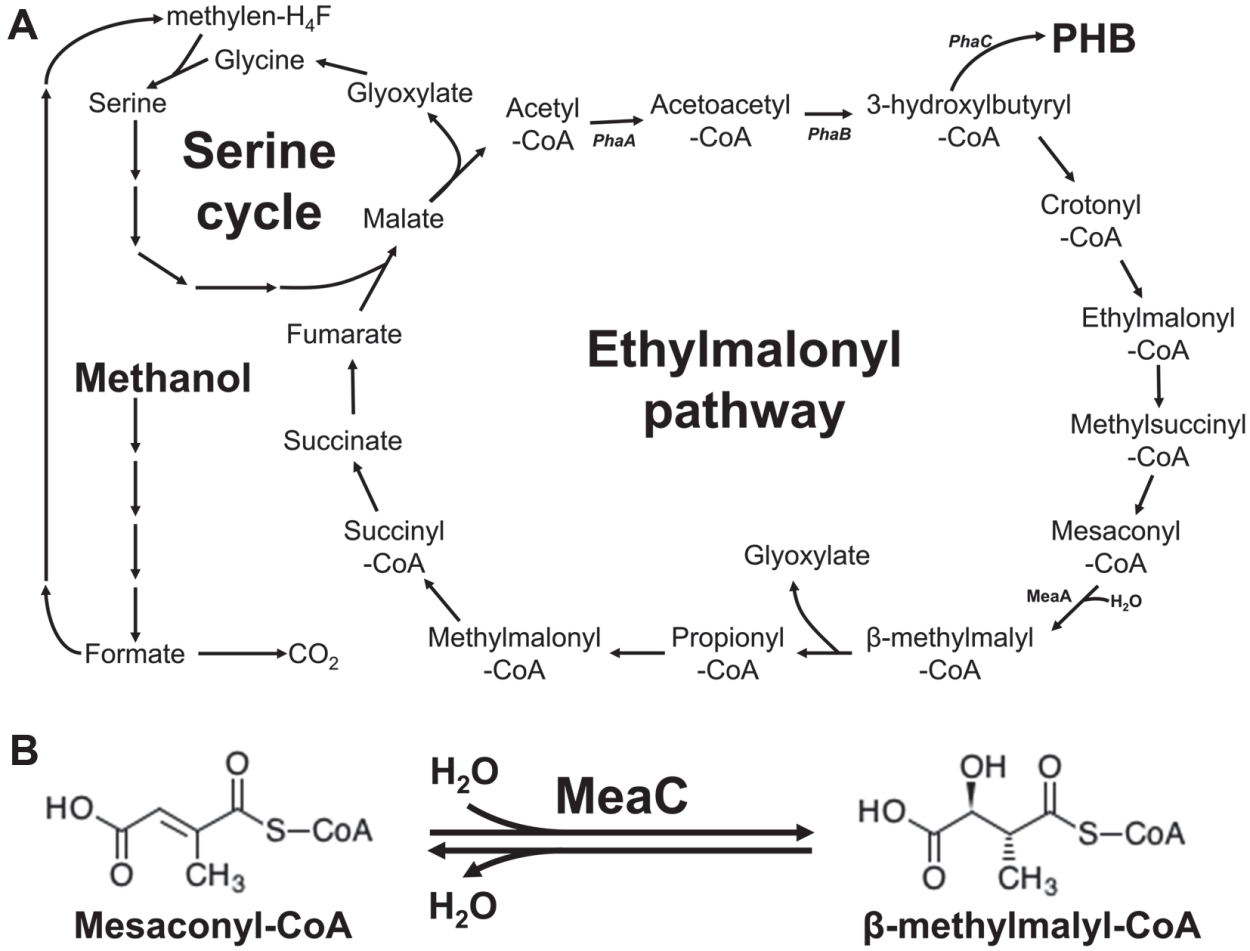
Ethylmalonyl-CoA pathway and mesaconyl-CoA dehydratase (MeaC) in *M. extorquens* CM4. (**A**) Ethylmalonyl-CoA pathway. Gene names are in bold italic. PhaA, β-ketothiolase; PhaB, NADPH-dependent acetyl-CoA reductase; PhaC, PHA synthase. (**B**) Catalytic reaction of MeaC by reversible dehydration/hydration.

*Methylorubrum extorquens* is facultative methylotroph that utilizes the serine cycle to assimilate C1 compounds such as methanol and supply C3 units for cell biosynthesis [7, 8]. It is well known that PHBs and PHAs are accumulated in many microorganisms as energy and carbon sources [9]. It is reported that *M. extorquens* also forms PHB granules and stores them like other PHB-producing bacteria [10,11]. Thus, as a model methylotroph, *M. extorquens* has been researched for understanding methylotrophy and producing value-added products from methanol. The ethylmalonyl-CoA pathway is central to the carbon metabolism of many α-roteobacteria, including *M. extorquens* [12]. The ethylmalonyl-CoA pathway is an anaplerotic reaction sequence essential for the assimilation of acetyl-CoA, which is necessary for methylotrophic growth (Fig. 1A). The condensation of two acetyl-CoA to acetoacetyl-CoA by β-ketothiolase (PhaA) and the reduction of acetoacetyl-CoA to (*R*)-3-hydroxybutyryl-CoA by NADPH-dependent acetyl-CoA reductase (PhaB) are shared with the PHA synthesis pathway [13]. Additionally, (*R*)-3-hydroxybutyryl-CoA can be polymerized to PHB by PHA synthase (PhaC). As an intermediate in the pathway, the downstream pathway comprises several CoA-activated mono- and dicarboxylic acids, such as crotonic, (2*R*)- and (2*S*)-methylsuccinic, as well as mesaconic acid, that are all interesting platform chemicals for the chemical industry [12].

**Fig. 2:**
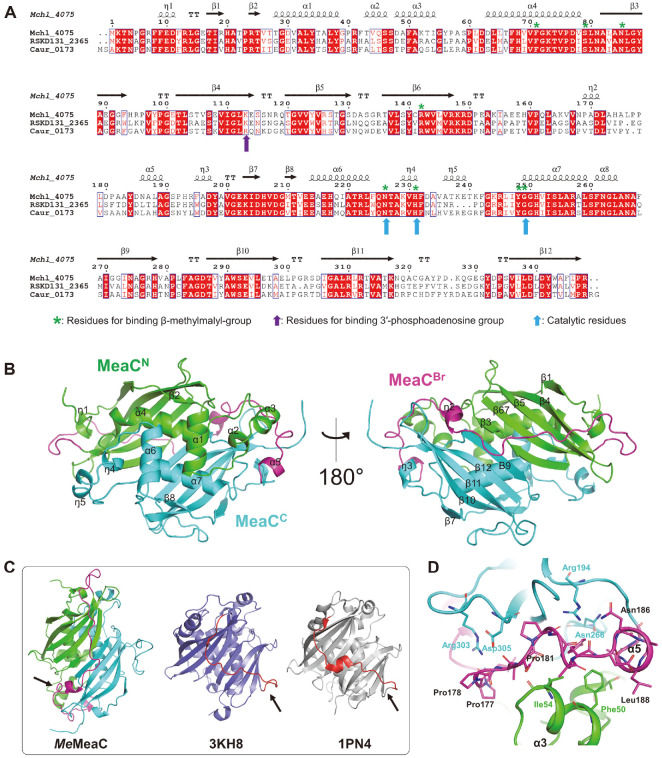
Sequence alignment and overall structure of MeaC. (**A**) Amino acid sequence alignment of *M. extorquens* MeaC with MeaC from *C. aurantiacus* and *R. sphaeroides*. The secondary structures are indicated above the sequence alignment. *M. extorquens* MeaC, Mch1_4075; *C. aurantiacus* MeaC, Caur_0173; *R. sphaeroides* MeaC, RSKD131_2365. (**B**) Overall structure of *Me*MeaC. MeaC^N^, MeaC^Br^, and MeaC^C^ are colored green, cyan, and magenta, respectively. (**C**) Comparison for bridge parts. Bridge parts of both MaoC-like hydratase from *P. capsici* (PDB code 3KH8) and 2-enoyl-CoA hydratase from *C. tropicalis* (PDB code 1PN4) are colored red. (**D**) Grasped MeaC^Br^ (residue 177 ~190) between MeaC^N^ and MeaC^C^.

MeaC functions as a mesaconyl-CoA hydratase, reversibly catalyzing mesaconyl-CoA into β-methylmalyl-CoA, by adding one water molecule (Fig. 1). Chistoserdova’s group identified that MeaC is involved in a step between methylsuccinyl-CoA and propionyl-CoA in *M. extorquens* [14]. The MeaC mutant accumulated β-hydroxybutyrate, butyrate, ethylmalonate, and methylsuccinate but not β-hydroxyisobutyrate, methylmalonate, or succinate when meaC mutant was grown with ^14^C-label carbon without unlabeled methanol. Moreover, Fuchs’ group identified and characterized mesaconyl-CoA hydratase as a new enzyme of the enoyl-CoA hydratase family in *Chloroflexus aurantiacus* and *Rhodobacter sphaeroides* (*Cereibacter sphaeroides*) [15, 16]. They revealed that β-methylmalyl-CoA can be reversibly dehydrated to mesaconyl-CoA by *C. aurantiacus* MeaC (Caur_0173) and *R. sphaeroides* MeaC (RSKD131_2365) with 59.9% and 61.5% sequence identity to *M. extorquens* MeaC (Mchl_4075), respectively (Fig. 2A).

In spite of the progress in the study of MeaC, there have been no reports on the structure containing the substrate or its analogues in the active site so far. It is not easy to present the substrate specificity at the molecular level or reveal the molecular mechanism without a structure. Thus, in this study, we determined the crystal structure of MeaC from *Methylorubrum extorquens* CM4 (*Me*MeaC). From the structural information for the *Me*MeaC structure containing the analogue, we suggest the molecular function and mechanism, which will contribute to understanding ethylmalonyl-CoA pathway as well as the 3-hydroxypropionate cycle for autotrophic CO_2_ fixation.

## Materials and Methods

### Expression and Purification

*MeMeaC* (Mchl_4075) was amplified with forward and reverse primers designed as 5’-GCGCCATATGAA GACCAATCCGGGCCGCTTCTTC-3’ and 5’-ATATCTCGAGGCGCGGGATGAAGGCCCAATAGTC-3’, respectively. The amplified gene was cloned into pET30a expression vector using NdeI and XhoI restriction enzymes. The pET30a:*MeMeaC* was transformed into the *E. coli* strain BL21(DE3)-T1^R^. Cells were cultured in an LB medium containing kanamycin at 37ºC, until reaching an absorbance of 0.7 at 600 nm. After induction using 1.0 mM isopropyl β-D-1-thiogalactopyranoside (IPTG), cells were incubated for 18 h at 20ºC. Following harvest, the cell pellet was resuspended in A buffer (40 mM Tris-HCl, pH 8.0, 150 mM NaCl, and 5 mM β-mercaptoethanol) and disrupted by ultrasonication with a pulse. The cell debris was removed by centrifugation at 13,000 ×*g* for 1 h. The lysate was bound to Ni-NTA agarose (Qiagen, Germany) by gravity. After washing with lysis buffer containing 30 mM imidazole, the protein was eluted by using B buffer (40 mM Tris-HCl, pH 8.0, 150 mM NaCl, 300 mM Imidazole, and 5 mM β-mercaptoethanol). To improve purity for crystallization, the protein was further purified by size exclusion chromatography using a HiPrep 2.6/60 Sephacryl S-300 HR column (Cytiva, USA). The purified protein was concentrated to 68 mg/ml in a solution with the buffer A.

### Crystallization

Initial crystallization of the purified protein was carried out by using commercial screening solutions, including Index, PEG Ion Screen I and II (Hampton Research, USA), and Wizard Classic I and II (Rigaku Reagents, USA), as the sitting-drop vapor-diffusion method at 20°C. We mixed 1.0 μl of the protein solution with 1.0 μl of the reservoir solution and then equilibrated this against 500 μl of the reservoir solution. Then, we identified crystals at conditions of 0.2 M DL-malic acid (pH 7.0) or 0.2 M sodium malonate (pH 7.0) with 20% (w/v) PEG3350 precipitant. After adjusting protein concentration, we could obtain suitable crystals under 0.2 M DL-malic acid (pH 7.0) and 20% (w/v) PEG3350 in 1–3 days.

### Data Collection and Processing

A cryo-protectant containing 30% (v/v) glycerol in a reservoir solution was used for X-ray diffraction. The datasets of the native protein were collected at 100 K at the 7A beamline of the Pohang Accelerator Laboratory (PAL, Republic of Korea), using a Quantum 270 CCD detector (ADSC, USA). The best crystal diffracted to 1.95 Å resolution. The collected data was commonly indexed, integrated, and scaled using the *HKL2000* suite (HKL Research, USA). The crystal belonged to space group *P6_3_* with the following unit cell parameters: a = 79.224 Å, b = 79.224 Å, c = 91.008 Å, α = β = 90°, and γ = 120°. The data statistics are summarized in Table 1.

### Structure Determination and Refinement

The MeaC structure was determined by the molecular replacement method with the CCP4 version of MOLREP [17]. The crystal structure of the putative MaoC domain protein dehydratase from *Chloroflexus aurantiacus* J-10-fl (PDB code 4E3E) was used as a search model. The initial model building was automatically performed using *ARP/wARP*, and the final model was built by using the program *WinCoot* [18, 19]. The refinement was performed with *REFMAC5* in *CCP4* suite [20, 21]. The refined model was deposited in the Protein Data Bank with the PDB code 8HGN. Data collection and refinement statistics are given in Table 1.

### Size Exclusion Chromatography

Analytical size exclusion chromatography was performed using a Superdex 200 Increase 10/300 GL column (Cytiva, USA). MeaC protein sample of 1.0 ml at 1 mg/ml was equilibrated with 40 mM Tris-HCl, pH 8.0 and 150 mM NaCl. The molecular mass of *Me*MeaC was calculated using a calibration curve with four standard samples, including ferritin (440 kDa), aldolase (158 kDa), ovalbumin (44 kDa), and ribonuclease A (13.7 kDa).

## Results and Discussion

### Overall Structure of *Me*MeaC

To elucidate the molecular mechanism and substrate binding mode of *Me*MeaC, we determined the crystal structure of the enzyme at a 1.95 Å resolution with *R*_work_ = 20.9% and *R*_free_ = 27.8%, respectively. The crystal structure of *Me*MeaC revealed that the enzyme belongs to the MaoC-like dehydratase domain superfamily, which consists of two central helices and a curved eleven-stranded antiparallel β-sheet (Fig. 2B). *Me*MeaC is composed of an N-terminal subdomain (MeaC^N^, residue 1~147), a C-terminal subdomain (MeaC^C^, residue 191~348), and a bridge (MeaC^Br^, residue 148~190) linking the two subdomains. Each subdomain with a hotdog topology is built of a curved, five-stranded antiparallel β-sheet (MeaC^N^: β1-β4-β5-β6-β3 and MeaC^C^: β7-β10-β11-β12-β9) that wraps around the central helix (MeaC^N^: α4 and MeaC^C^: α7) [22]. The overall structure of *Me*MeaC is maintained by the interaction between MeaC^N^ and MeaC^C^ with extensive non-covalent contacts, including a long antiparallel β-sheet formed from β3-β9 (Fig. 2B). MeaC^Br^ connects MeaC^N^ to MeaC^C^ by crossing over the saddle side generated by their interactions. When we compared *Me*MeaC with MaoC-like hydratase from *Phytophthora capsici* (PDB code 3KH8) and 2-enoyl-CoA hydratase from *Candida tropicalis* (PDB code 1PN4), which are divided into three parts (MeaC^N^, MeaC^C^, and MeaC^Br^), we found that the connection by MeaC^Br^ differ from others (Fig. 2C). While other bridges head directly to the end of central β-sheet of a C-terminal subdomain, MeaC^Br^ passes over between α3 of MeaC^N^ and the loop of β10–β11 in MeaC^C^. Particularly, residue 177 ~190 on MeaC^Br^ is grasped by Arg194, Asn266, Asg303, Asp305 on MeaC^N^, and Thr43, Ile54, Phe50 on MeaC^C^ (Fig. 2D). This grip enhances the MeaC^N^–MeaC^C^ interaction, which could contribute to maintaining the overall scaffold of *Me*MeaC.

**Fig. 3:**
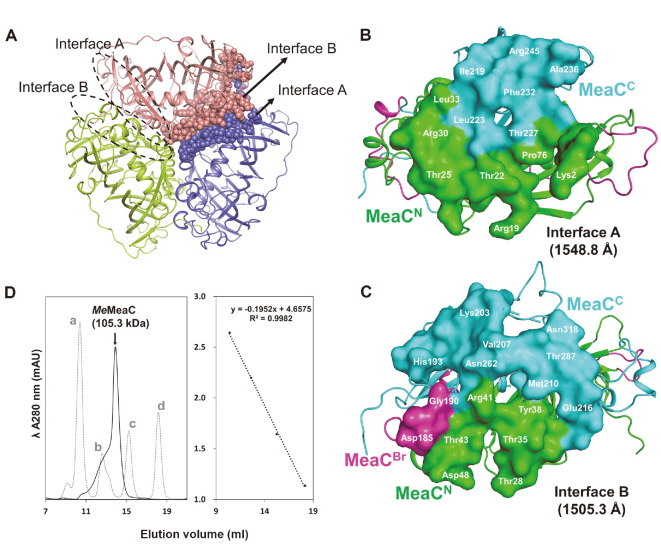
Trimerization of *Me*MeaC. (**A**) Trimer of *Me*MeaC. Each chain is depicted as a cartoon in salmon, light-green, and lavender-blue, respectively. (**B**) Interface A. MeaC^N^ and MeaC^C^ regions are displayed with green and cyan surfaces, respectively. (**C**) Interface B. MeaC^Br^ region is shown with violet surface. (**D**) Analysis of size exclusion chromatography of *Me*MeaC. *Me*MeaC and reference proteins are drawn as line and dash, respectively. a, ferritin (440 kDa); b, aldolase (158 kDa); c, ovalbumin (44 kDa); d, Ribonuclease A (13.7 kDa).

Although one molecule is present in the asymmetric unit of the crystal structure, the *P6_3_* crystallographic symmetry operation of *Me*MeaC revealed that the enzyme forms a trimer. PDBePISA (Proteins, Interfaces, Structures, and Assemblies) also suggested that *Me*MeaC forms a trimer [23]. The trimer is assembled by the interaction between interfaces A and B with buried surface areas of 1548.8 Å and 1505.3 Å, respectively (Figs. 3A-3C). Taking account of the wide area of interface A and B in the trimer assembly, we speculated that *Me*MeaC could stably maintain the trimer in solution. When the size-exclusive chromatography experiment was performed, the molecular weight of *Me*MeaC was calculated as 105.3 kDa, similar to a trimer, which confirms the trimerization in solution (Fig. 3D).

### CoA Binding Site of *Me*MeaC

**Fig. 4:**
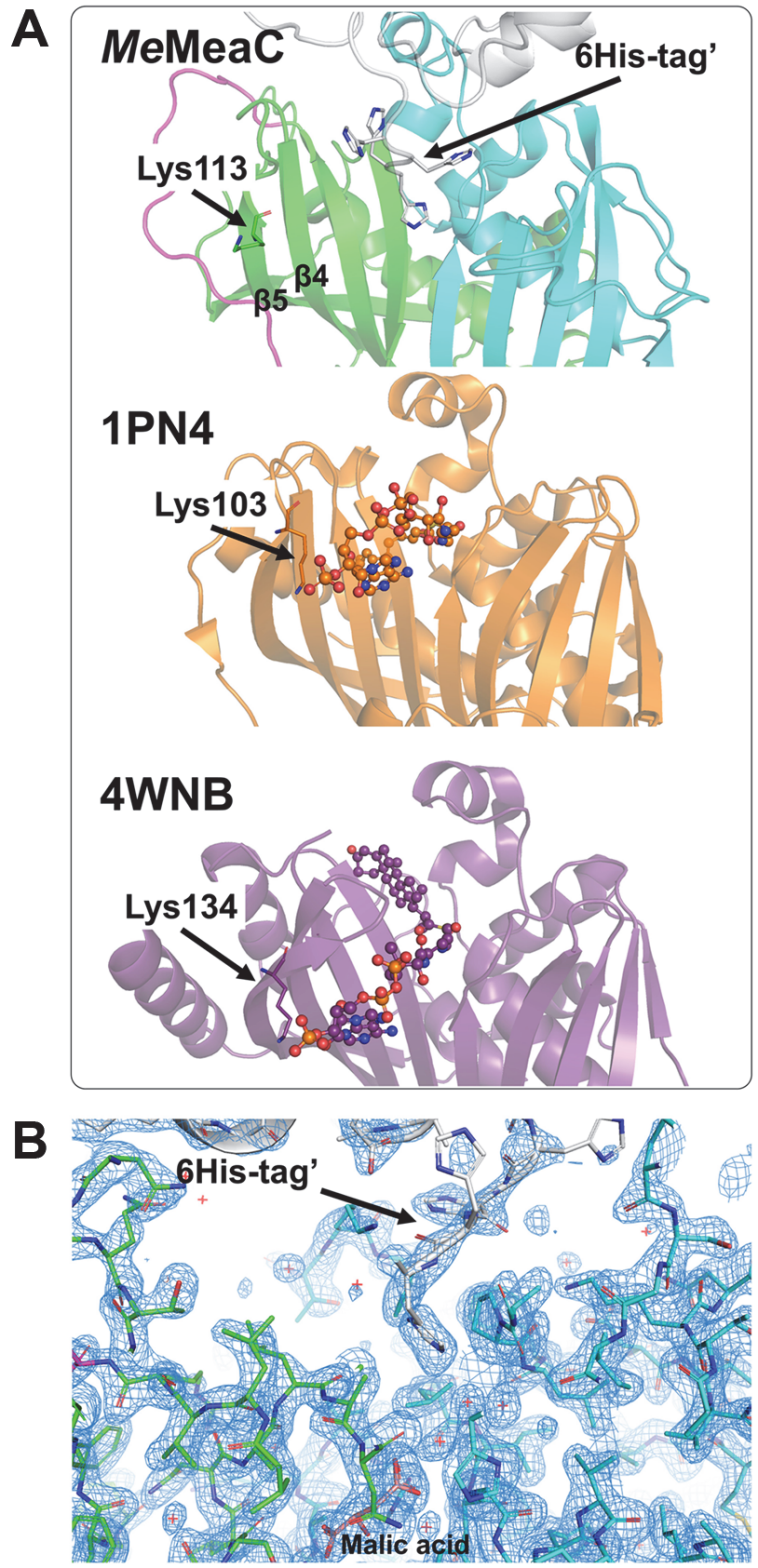
CoA binding site in *Me*MeaC. (**A**) Juxtaposition of superposed structures. Each structure overlapped is stacked in a row. CoA derivatives are drawn as balls and sticks. 2-enoyl-CoA hydratase from *C. tropicalis* (PDB code 1PN4) and ChsH1- ChsH2 complex in *M. tuberculosis* (PDB code 4WNB) are colored orange and purple, respectively. (**B**) *2Fo – Fc* map of intruded 6His-tag. Map is contoured at 1.5 δ. Protein molecules are drawn as sticks and the white stick implies the 6His-tag of another chain by crystal packing.

To elucidate the substrate binding mode of *Me*MeaC, we attempted to determine the complex structure with the CoA molecule, however, both soaking and co-crystallization experiments failed. Alternatively, superposition of the structure of *Me*MeaC with those of enoyl-CoA hydratases in complex with CoA (PDB codes: 1PN4 and 4WNB) revealed that the 6xHis-tag of *Me*MeaC is positioned in the vicinity of the CoA binding site and interferes with the binding of CoA into the enzyme (Figs. 4A and 4B). Among structures of MaoC-like enoyl-CoA hydratases, 2-enoyl-CoA hydratase from *C. tropicalis* (PDB code 1PN4) and ChsH1-ChsH2 complex in *Mycobacterium tuberculosis* (PDB code 4WNB) contain (3*R*)-hydroxydecanoyl-CoA and 3-oxo-4-pregnene-20-carboxyl-CoA (3-OPC-CoA), respectively [24, 25]. Superposition of the *Me*MeaC structure with 1PN4 (RMSD = 5.9 Å) and 4WNB (RMSD = 7.7 Å) allowed us to identify the binding mode of CoA in *Me*MeaC. The CoA binding site might be formed between β4–loop–β5 and β10–loop–β11 on the saddle side formed by the combination of MeaC^N^ and MeaC^C^, and particularly, the 3’-phosphoadenosine-moiety might be positioned near the β4-loop-β5 in *Me*MeaC (Fig. 4A). The crystal structures of *Me*MeaC, 2-enoyl-CoA hydratase from *C. tropicalis* (PDB code 1PN4) and ChsH1-ChsH2 complex in *M. tuberculosis* (PDB code 4WNB) show that conserved lysine is commonly located on β4–loop–β5 of their structure (Fig. 4A). The Lys103 of 1PN4 and Lys134 of 4WNB interact with the 3¢-phosphoadenosine-moiety of CoA. Thus, considering that Lys113 is also located at the corresponding position in *Me*MeaC (Figs. 2A and 4A), these observations imply that Lys113 is a crucial residue for stabilization of the 3¢-phosphoadenosine-moiety of CoA near the β4-loop-β5 in MaoC-like enoyl-CoA hydratases, including *Me*MeaC.

### Stabilization Mode of the β-Methylmalyl-Moiety

**Fig. 5:**
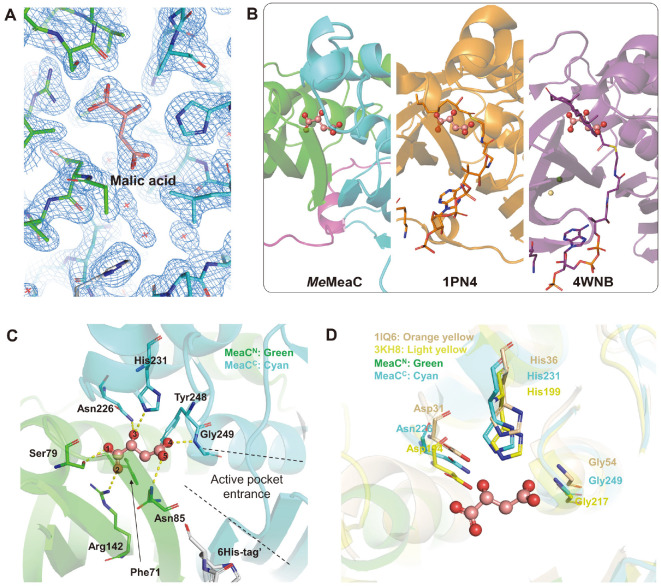
Active pocket and catalytic residues in *Me*MeaC. (**A**) *2Fo – Fc* map of the malic acid (contoured at 1.5 δ). Salmon stick indicates malic acid in the active pocket. (**B**) Juxtaposition of superposed structures with each placed side by side. CoA derivatives are described as sticks with their own colors. (**C**) Residues in the putative active site. The malic acid is displayed as balls and sticks with salmon color. Polar contacts are indicated as yellow dashes. Numbers on balls mean the numbering for oxygens in the malic acid. (**D**) Overlapping putative catalytic residues. Putative catalytic residues are described as sticks. MaoClike hydratase from *P. capsici* (PDB code 3KH8) and (*R*)-specific enoyl coenzyme A hydratase from A. caviae (PDB code 1IQ6) are shown in light yellow and gold color, respectively.

In our current structure of *Me*MeaC, we observed that a malic acid molecule, which is used as a main precipitant for the crystallization, is bound at the active site (Fig. 5A). When we superpose the structure of *Me*MeaC with 1PN4 and 4WNB, the malic acid molecule in *Me*MeaC was located at the position similar to (3*R*)-hydroxydecanoyl- and 3-OPC-moiety bound in 1PN4 and 4WNB, respectively (Fig. 5B). Moreover, because the structure of malic acid is quite similar to the β-methylmalyl- and mesaconyl-moiety of β-methylmalyl-CoA and mesaconyl-CoA, respectively, we suspect that the β-methylmalyl- and mesaconyl-moiety might be stabilized in a mode similar to malic acid in the *Me*MeaC. The binding site of the malic acid molecule is formed at the interface between MeaC^N^ and MeaC^C^ (Fig. 5C). The conserved residues, such as Phe71, Ser79, Asn85, Arg142, Asn226, His231, Tyr248, and Gly249, were located at the malic acid binding site (Figs. 2A and 5C). Based on the spatial position of these residues and the binding mode for malic acid, it could be inferred that they are required for holding the β-methylmalyl- and mesaconyl-moiety and performing the enzyme activity. The crystal structure of *Me*MeaC shows that these residues form polar contact and hydrophobic interaction for binding of the malic acid in the active site (Fig. 5C). Oxygen of Ser79 sidechain and nitrogen of Arg142 sidechain make hydrogen bonds to O1 and O2 of malic acid with 2.6 Å and 2.8 Å distances. Nitrogen atoms of Asn85 and Gly249 backbones form hydrogen bonds to O4 and O5 of malic acid with 2.6 Å and 3.0 Å distances. O3 of malic acid interacts with nitrogen atoms of sidechains of Asn226 and His231 as hydrogen bonds of 2.6 Å and 3.0 Å distances. There are hydrophobic interactions between Phe71, Tyr248, and the carbon backbone of the malic acid, which contribute to stabilizing binding β-methylmalyl-moiety at the active site.

Amongst these interactions, it is speculated that the double hydrogen bonds of Asn226–O3–His231 could play a pivotal role in the substrate recognition and the enzyme reaction. Taking into account that a β-methylmalyl-CoA has an additional hydroxyl group at the counterpart of O3 of the malic acid and is not at a mesaconyl-CoA, strong interaction of Asn226–O3–His231 could make the binding of a β-methylmalyl-moiety more stable, which contributes to improving the specificity. Moreover, when the *Me*MeaC structure is overlapped on MaoC-like hydratase from *P. capsici* (PDB code 3KH8, RMSD = 4.2 Å) and (*R*)-specific enoyl-CoA hydratase from *Aeromonas caviae* (PDB code 1IQ6, RMSD = 3.9 Å) [26, 27], Asn226 and His231, known as catalytic residues in other MaoC-Like hydratases, are also positioned in *Me*MeaC (Fig. 5D), suggesting that *Me*MeaC catalyzes the enzyme reaction a similar mode to other MaoC-like hydratases. Therefore, assuming that *Me*MeaC has the reaction mechanism via the concerted transition state like the proposed mechanism of 2-enoyl-CoA hydratase from *C. tropicalis* (PDB code 1PN4) [24], Asn226 and His231 might function as activating single water molecules for the hydration/dehydration reaction.

In summary, we determined the crystal structure of *Me*MeaC with a 1.95 Å resolution. Compared to the other structures of MaoC-like hydratases, the active pocket and CoA binding sites could be identified. The malic acid molecule bound in our structure assists in identifying the binding mode of the functional-moiety of the substrate. However, since it was hard to obtain the substrates, we were not able to measure in vitro activities for the mutant, which could solidify the molecular mechanism for the enzyme reaction and the substrate specificity. In the future, if the mutant activities are measured through in vivo and in vitro approaches, our structural analysis of *Me*MeaC will contribute to expanding the comprehension of the ethylmalonyl-CoA pathway and the 3-hydroxypropionate cycle by revealing the molecular mechanism for the reaction and substrate recognition of *Me*MeaC.

Methylotrophs such as *M. extorquens* utilize the ethylmalonyl-CoA pathway. *M. extorquens* is the best-studied methylotroph [8, 28-30] and is also the best platform for methanol-based biotechnology [31]. Considering that each intermediate in the ethylmalonyl-CoA pathway is a potential starting point for designing new pathways for producing high value-added chemicals [12], the understanding of meaC derived from this study may help in developing various material production methods using methanol based on *M. extorquens* in the future.

## Figures and Tables

**Table 1:** Data collection, phasing and refinement statistics of *Me*MeaC.

	*Me*MeaC + malic acid
Data collection	
Wavelength (Å)	0.979
Space group	*P6_3_*
Cell dimensions	
a, b, c (Å)	79.22, 79.22, 91.00
α, β, γ (°)	90.00, 90.00, 120.00
Resolution (Å)	50.00-1.95 (1.98-1.95)
*R_sym_* or *R_merge_* (%)	10.8 (24.8)
*I / σ (I)*	57.6 (10.9)
Completeness (%)	98.5 (93.4)
Redundancy	6.9 (4.1)
Refinement	
Resolution (Å)	32.10-1.95
No. reflections	23330
*R*_work_ / *R*_free_ (%)	20.9/27.8
No. atoms	2,817
Protein	2,718
Ligand/ion	9
Water	90
*B*-factors	32.8
Protein	35.5
Ligand/ion	31.9
Water	30.8
r.m.s. deviations	
Bond lengths (Å)	0.009
Bond angles (°)	1.697
PDB ID	8HGN

*Values in parentheses are for highest-resolution shell.
